# Structural validation of a brief, multidimensional measure of psychological flexibility and inflexibility in adolescence

**DOI:** 10.1186/s40359-025-03937-w

**Published:** 2026-01-10

**Authors:** Jakob Langenskiöld, Pekka Räsänen, Prince Das Adhikary, Rosa Salmela, Mikko-Jussi Laakso, Katarina Alanko

**Affiliations:** 1 Turku Research Institute for Learning Analytics, Faculty of Science, University of Turku, Turku, Finland; 2 Department of Pediatric Neurology, Epilepsia Helsinki, HUS Helsinki University Hospital, Helsinki, Finland

**Keywords:** Psychological flexibility, Psychological inflexibility, Children and adolescents, Acceptance and commitment therapy

## Abstract

**Background:**

Psychological flexibility and inflexibility (PF/PI) are increasingly targeted in clinical and preventive interventions as processes relevant to both flourishing and distress. However, brief multidimensional measures that assess both constructs and are developmentally appropriate for children and younger adolescents remain scarce. This study investigated the dimensionality of PF/PI in early and mid-adolescence, and conducted a preliminary structural validation of a brief questionnaire for potential use in school settings.

**Methods:**

Data were drawn from a cross-sectional sample of 1,289 Finnish lower secondary school students in grades six, eight, and nine. Eighteen items adapted from the Children’s Psychological Flexibility Questionnaire (CPFQ) were administered before a digital mathematics assessment. Both exploratory (EFA) and confirmatory factor analysis (CFA) with tests of measurement invariance were conducted using a split sample approach. Internal consistency was evaluated using alpha and omega coefficients, and average inter-item correlations.

**Results:**

The iterative item retention process resulted in a three-factor nine-item solution (CPFQ-9) that met predefined psychometric criteria and was replicated with CFA. The model included two modestly correlated PF factors (1) committed action with awareness, 2) acceptance and defusion) and one largely independent PI factor (3) self-judgment and fusion). Configural, metric and partial scalar invariance were supported across grade and gender. Subscale internal consistency were questionable to borderline acceptable but average inter-item correlations were within recommended ranged for shorter scales.

**Conclusions:**

Findings suggest that a brief, multidimensional measure can capture developmentally relevant flexibility- and inflexibility-related processes in early and mid-adolescence. Further work is needed to establish convergent, divergent, and predictive validity, test-retest reliability, and applicability in younger age groups and contexts, before the CPFQ-9 can be considered a robustly validated, developmentally sensitive measure.

**Supplementary Information:**

The online version contains supplementary material available at 10.1186/s40359-025-03937-w.

## Background

Psychological flexibility (PF) and its counterpart, psychological inflexibility (PI), are key constructs in acceptance and commitment therapy (ACT) [1] that synthesize processes for relating to internal psychological experiences, and behavior change. PF is defined as the willingness to engage with unwanted thoughts and feelings while behaving in ways that are consistent with one’s values, whereas PI refers to the rigid dominance of psychological reactions over chosen values and contingencies in guiding behavior [2].

Although the literature on associations between PF/PI and relevant mental health indicators is more limited for children and adolescents, a growing body of evidence has begun to emerge. PF skills have been linked to better overall functioning, prosocial behavior, well-being, reduced stress, and positive behavior change [3–6]. Conversely, PI has been associated with a range of negative outcomes, including various forms of psychopathology [7].

Promoting PF and preventing PI may therefore serve as a buffer for adaptation during childhood and adolescence in supporting developmental tasks such as identity formation, the identification of personal values, and the establishment of self-regulatory capacities [3]. That the ACT model includes specific skills training procedures aimed at developing PF and preventing PI provides further reason for why PF and PI have increasingly become targets in school-based mental health – promoting efforts for children and adolescents [8–11].

Valid and reliable measurement of PF and PI in children and adolescents, that is feasible in the school setting, is therefore essential, both for identifying protective and risk factors for healthy development, for screening and research purposes, and for monitoring mechanisms of change in PF/PI – focused interventions. To serve these purposes, measures must (1) adequately capture the multidimensional nature of PF/PI [12, 13]; (2) be sufficiently brief to allow for routine administration in classroom settings, where time constraints due to school-day logistics, limited time for psychosocial programs, and the use of multiple instruments are common [14, 15], and (3) employ developmentally appropriate items that are comprehensible and psychometrically adequate across different age groups [7, 16].

### The hexaflex model and structural controversy

The most commonly used structural representation of PF/PI is the “hexaflex” model [1, 17], that comprise six interrelated subprocesses, each with a PF and PI variant: acceptance vs. experiential avoidance, cognitive defusion vs. fusion, present-moment awareness vs. inflexible attention, self-as-context vs. self-as-content, values vs. disruption of values, and committed action vs. inaction/impulsivity (see Table 1).

There is, however, a discrepancy between the hexaflex model, and empirical findings regarding the structural organization of PF/PI [12]. First, PF and PI do not appear to represent opposite ends of a single continuum, rather, they function as distinct constructs [7, 12, 18, 19]. Second, PF and PI are frequently operationalized using unidimensional instruments [20–22]. In the child and adolescent literature, this is most evident in the widespread use of the Avoidance and Fusion Questionnaire for Youth (AFQ-Y) [21]. Even though the AFQ-Y consists of exclusively PI items that load onto a single factor, interpretating high AFQ scores as a proxy for low PF, and vice versa, remains an occurring practice in the literature [23–25]. Third, multidimensional instruments used with younger populations tend to collapse into more parsimonious structures than the six-process hexaflex. These include two-factor models in which PF and PI form distinct but correlated subdomains [12], or three-factor models that align theoretically with the so-called “triflex” model [13], which integrates the hexaflex processes into to the three broader functional domains; open (acceptance and defusion), active (values and committed action) and aware (present-moment awareness and self-as-context).

**Table 1:** Overview of the psychological flexibility and inflexibility processes in the hexaflex model

Process	Psychological Flexibility (PF)	Psychological Inflexibility (PI)
Acceptance vs. Experiential Avoidance	Willingness to experience all types of thoughts and feelings.	Attempts to avoid and suppress unwanted thoughts and feelings.
Cognitive Defusion vs. Cognitive Fusion	Seeing thoughts as just thoughts, rather than absolute truths.	Interpreting thoughts literally and allowing them to control behavior.
Present-Moment Awareness vs. Inattention	Flexible and mindful attention to the present moment.	Distraction and entanglement with past or future events.
Self-as-Context vs. Self-as-Content	Viewing oneself as greater than transient thoughts, feelings, or roles.	Being overly attached to rigid self-stories or labels.
Values vs. Disruption of Values	Clarifying what truly matters to oneself.	Lack of clarity or awareness about what truly matters.
Committed Action vs. Inaction	Engaging persistently in behaviors that align with one’s values.	Avoiding action, acting impulsively, or acting contrary to one’s values.

### Brief multidimensional PF/PI measurement in children and adolescents

In addition to the lack of consensus regarding the structural organization of PF/PI, and how it may differ across developmental stages, there remains a shortage of briefer, validated multidimensional instruments that assess both PF and PI processes, and are appropriate for younger adolescents and children. Since the promotion of PF and the reduction of PI appear to represent distinct intervention targets [26], linked to different outcomes of wellbeing [27], it is essential to assess both constructs in order to capture the full scope of PF/PI – focused interventions [26].

A promising development is the youth version of the CompACT (CompACT-Y) [13], which has been preliminary validated in a sample of adolescents aged 13–18 (M = 16.25). Evidence for its structural validity remains, however, preliminary, relying on a single study that employed exploratory factor analysis (EFA) without confirming the model in a separate sample or testing measurement invariance across relevant developmental subgroups, such as age and gender. With 19 items in its final form, the CompACT-Y is also relatively lengthy for routine use in school-based interventions, where brevity and feasibility are essential.

An instrument that offers a promising foundation for developing a multidimensional, shorter PF/PI form suitable for routine classroom assessment across different stages of development is the Children’s Psychological Flexibility Questionnaire (CPFQ) [28]. The original CPFQ consists of 24 items that assess both PF and PI variants of each hexaflex process, written in child-friendly language, with the aim to be suitable for a wider range of young people. Evidence of convergent validity and developmental appropriateness has been demonstrated in samples ranging from 10-year-old children to adults over 50 [29, 30]. This age appropriateness, together with its representative coverage of hexaflex processes, provides a strong basis for investigating the dimensionality of PF/PI in younger populations, and constructing a shorter multidimensional instrument.

Utilizing a large sample (*N* = 1289) of Finnish students from grades six (typically aged 12–13 years), eight (typically aged 14–15 years), and nine (typically aged 15–16 years), this study has two aims, informing both theory and practice: (1) to investigate the dimensionality of PF/PI in early and mid-adolescence, contributing to the discussion regarding the structure of PF/PI in younger populations; and (2) to develop and structurally validate a brief, multidimensional, and developmentally sensitive PF/PI measure based on 18 items, derived from the Children’s Psychological Flexibility Questionnaire (CPFQ).

### The present study

We employ a cross-sectional, split-sample design to conduct the structural validation with the 18 items as the starting point: exploratory factor analysis (EFA) is first used to identify a factor structure and employ an item reduction process that optimally balances psychometric rigor and conceptual coherence, followed by confirmatory factor analysis (CFA) and tests of measurement invariance (MI) across grade levels and gender. Additionally, internal consistency is estimated.

To our knowledge, this is the first study to conduct a structural validation including both EFA and CFA, as well as MI testing, of a multidimensional PF/PI measure specifically developed for children and adolescents. Because CPFQ items have not previously undergone factor analysis, and given that existing youth PF/PI measures have yielded varying structural organizations, an exploratory approach is as an appropriate starting point for identifying the most proper factor structure of a brief CPFQ-derived instrument.

## Methods

### Participants

The sample consisted of 360 students from Finnish-speaking lower secondary schools, 571 sixth-graders (typically aged 12–13 years), 332 eighth- graders (typically aged 14–15 years) and 386 ninth-graders (typically aged 15–16 years). Of these, 668 (51.8%) were boys and 621 (48.2%) girls.

To ensure a rigorous validation process, the total sample was divided into two subsamples, of which the first was used to identify the latent structure of the item set through exploratory factor analysis (EFA), and the second was reserved for confirmatory factor analysis (CFA) and measurement invariance testing (MI).

The EFA subsample consisted of 360 students, including 166 eighth-graders and 194 ninth-graders (52% boys). The CFA subsample (*n* = 929, 51.8% boys) included the remaining eighth- (*n* = 166) and ninth-grade (*n* = 192) students together with the complete set of sixth-grade students (*n* = 571).

The choice not to include sixth graders in the EFAs was to maximize the power of the CFAs with MI testing and to investigate whether the identified factor structure is generalized to an earlier stage of development.

### Data collection

The study followed the Finnish law, the ethical guidelines of the Finnish National Board on Research Integrity (TENK), and the Declaration of Helsinki. Research permissions were obtained from the participating municipalities. Guardians were informed, and all the students participated anonymously and voluntarily. A passive consent procedure was used, allowing students to decrease the usage of their data.

Data were collected in April 2025, as part of the DigiEva project, which assessed mathematical abilities at the end of the school year across grades 3, 6, 8, and 9 in Finnish- and Swedish-speaking schools in Finland.

Assessments were completed electronically via the ViLLE digital platform [31], with the 18 items embedded in a set of socio-emotional background questionnaires completed prior to the test tasks.

### Measure

#### Children’s psychological flexibility Questionnaire – 18 item version

Our items were derived from The Children’s Psychological Flexibility Questionnaire (CPFQ) [28], which is a 24-item self-report measure that covers each process in the hexaflex model: present moment awareness, acceptance, cognitive defusion, self-as-context, values, and committed action. Each subscale includes both positively and negatively worded items and thus hold the potential to assess both PF and PI variants of each hexaflex process, even though the original CPFQ scoring procedure aggregates subscale scores into a single overarching PF score.

Each subscale contains four items (two positively and two negatively worded). Responses are recorded on a five-point Likert scale, ranging from “never” (0) to “always” [4]. Subscale scores are summed to form the total PF score, ranging from 0 to 96.

Due to time constraints before the mathematics test, the item pool was reduced to 18 items, selecting three items per subprocess to subscale to ensure complete coverage of both PF and PI variants of each hexaflex process and since three items per dimension is often regarded as the minimum to achieve stable factor modeling. This pragmatic reduction also aligns with one of the study aims, which is to develop a brief PF/PI instrument suitable for routine use.

The item set consisted of nine positively worded items and nine negatively worded items. For the statistical analyses, the negatively worded items were reverse scored, such that higher scores indicate low levels of PI, whereas higher scores on the positively worded items indicate higher PF. The total score of the 18-item set ranges from 0 to 72.

The selection of the 18 items was conducted by two of the authors, both trained clinical psychologists. For each subprocess, one item was removed, selecting items whose content showed greatest overlap with another item in the subscale or whose wording was deemed as less comprehensible compared to other items in the subscales. See Appendix A for the item order of the original CPFQ 24-item pool and the inclusion/exclusion decisions made for the 18-item version.

When constructing the reduced form, three items were simplified for clarity: 1) “If I lose I try again right away to do better” to “If I fail, I try again right away to do better”; 2) I notice my thoughts and feelings but that is not me” to “I notice my thoughts and feelings”; and 3) “I miss seeing stuff happen or hearing what people say” to “Sometimes I don’t notice what’s happening or what people say”. The item order was adjusted based on comprehension difficulty, progressing from easier to more challenging, as judged by the authors with backgrounds in clinical psychology.

The translation into Finnish was done through collaboration between two authors fluent in both English and Finnish. To ensure semantic consistency in item content, a back-translation procedure was employed, with a third author translating the items back into English. The back-translation was consistent with the original English items without any problematic deviations appearing. See Appendix B for the output of the translation process.

### Data analysis

#### Descriptive statistics

Means and standard deviations for each item and the total score of the full 18-item scale were calculated separately by grade level and gender.

#### Exploratory factor analysis

To explore the underlying structure of the items, we conducted exploratory factor analysis (EFA) using principal axis factoring (PAF), using direct oblimin rotation, as the factors were assumed to be correlated [32, 33]. Although CPFQ responses are technically ordinal, we treated them as continuous and used Pearson’s correlation instead of polychoric correlation, which is an acceptable procedure for scales with five or more response categories [33–35]. PAF was chosen for its robustness to nonnormality and its suitability for uncovering latent structure (32,33).

Prior to analysis, data suitability was assessed through univariate skewness and kurtosis (thresholds ≥ 2.0 and ≥ 7.0), histograms and Q-Q plots. Bartlett’s test of sphericity and the Kaiser‒Meyer‒Olkin test (KMO) [36] were used to confirm factorability, with a KMO ≥ 0.50 required [33]. The determinant of the correlation matrix was checked for multicollinearity.

Following best-practice guidelines [33], we determined the number of factors using multiple methods and made an integrative decision based on scree plot analysis [37], parallel analysis [38] and the Minimum Average Partial (MAP) [39] test. When these methods indicated different factor structures, our decision was based on a compromise between the alternatives, taking into account parsimony, factor stability (a minimum of three items per factor) [33], and the communalities among the items assigned to each factor.

Item retention decisions were made through iterative rounds in which items were flagged for removal based on four criteria. Three criteria (C1-C3) regarded factor loadings, following Howard’s “.40,.30,.20 rule” [40] which integrates best-practice recommendations by applying cut-offs of ≥ 0.40 for primary loadings (C1), < 0.30 for secondary loadings (C2), and a minimum difference of 0.20 between the two (C3). In addition, a fourth criterion (C4) was applied, specifying a minimum acceptable communality of 0.30.

In each round, the item that showed the most severe violation of the criteria was deleted, with concurrent violations of C1 and C4 considered most severe, followed by an evaluation of the number and magnitude of cross-loadings. Once the model had been refined by removing psychometrically unacceptable items, theoretical considerations guided further decisions regarding borderline-performing items. These considerations included the item’s conceptual alignment with PF/PI theory and potential content overlap with other, better-performing items. The refinement process continued until a satisfactory solution was achieved that reached our psychometric criteria, balanced with conceptual coherence.

The internal consistency of the factors was assessed using Cronbach’s alpha [41] and McDonald’s omega [42].

#### Confirmatory factor analysis

We continued to treat the variables as continuous due to the presence of five response categories [35], conducting the CFAs with maximum likelihood estimation. Item-level missingness, univariate and multivariate normality, were assessed to determine the suitability of the data and whether a robust maximum likelihood estimation method was required. Missing data were handled using full information maximum likelihood (FIML).

Following established guidelines [43], model fit was evaluated using Comparative Fit Index (CFI), Standardized Root Mean Square Residual (SRMR), Root Mean Square Error of Approximation (RMSEA) and the Tucker-Lewis Index (TLI), applying the following cutoffs for acceptable fit, found in the literature [44, 45]: CFI > 0.95 (good fit) or > 0.90 (acceptable fit), TLI > 0.95 (good fit) or > 0.90 (acceptable fit), SRMR < 0.08, and RMSEA < 0.06 (good fit) or < 0.07 (acceptable fit). The Chi-Square test of model fit is also reported with a statistical non-significant (*p* >.05) result indicating good fit. However, because this test is highly sensitive to sample size, it was not used as a primary indication of model fit in our analyses [44]. Modification indices were examined to determine whether conceptually justifiable correlations between residuals could be included to improve model fit.

Internal consistency was estimated with Cronbach’s Alpha and McDonald’s omega. In addition, average inter-item correlations (AIIC) were calculated for each factor, as this estimate is by some scholars considered more useful than Cronbach’s alpha for short scales [46]. According to guidelines in the literature, an AIIC between 0.15 and 0.50 indicates an appropriate balance between internal homogeneity and lack of item redundancy [46].

#### Measurement invariance

Configural, metric, and scalar invariance were tested across grade levels and gender. In the configural invariance model, all parameters were freely estimated across groups. Metric invariance was tested by constraining factor loadings, and model fit indices were compared to the configural invariance model. The scalar invariance model further constrained both factor loadings and item intercepts, with model fit indices compared to the metric invariance model. As noted in Putnick and Bornstein’s state-of-the-art summary of guidelines [47], the literature lacks universally accepted criteria for determining acceptable model fit when comparing different levels of invariance. We therefore followed the guidelines found in the PF/PI literature [48], according to which at least two of the following criteria must be met to support invariance: ΔCFI and ΔTLI ≤ 0.01, and ΔRMSEA ≤ 0.015.

Descriptive statistics, EFA, CFA and MI tests (configural, metric, and scalar invariance models) were conducted using the jamovi software [49]. Simulated eigenvalues, the MAP test, partial scalar invariance models, the path diagram, and average inter-item correlations were generated in R, using the psych [50], lavaan [51], semPlot [52], and semTools [53] packages.

## Results

### Descriptive statistics

Item and order and descriptive statistics (M, SD) for each item and for the total score, stratified by age and gender using the full sample, are presented in Table 2.

### Exploratory factor analysis

#### Missing data and descriptive statistics

Complete data on all 18 items were available for 315 participants (87.5%). Item-level missingness was low (0–7 per item) and Little’s MCAR indicated that the data were consistent with MCAR (X^2^ [317] = 309.699, *p* =.605), justifying pairwise deletion.

#### Evaluation of data suitability for factor analysis

Univariate skewness and kurtosis indicated that no items exceeded thresholds for problematic non-normality. While histograms showed some deviations, factor analysis was conducted using Pearson’s correlations, due to principal axis factoring’s robustness to moderate nonnormality, and the large sample size.

Bartlett’s test of sphericity was significant (*P* <.001) and the Kaiser-Meyer-Olkin measure was 0.75 (item range 0.54–0.81), exceeding the recommended 0.50 threshold and supporting the suitability of the data for factor analysis.

**Table 2:** Item order in the 18- CPFQ item set and descriptive statistics

Item	Grade 6 Girls	Grade 6 Boys	Grade 8 Girls	Grade 8 Boys	Grade 9 Girls	Grade 9 Boys
1. I try really hard every day.	3.07 (0.78)^a^	2.99 (0.83)	2.82 (0.76)	2.50 (0.88)	2.91 (0.75)	2.63 (0.83)
2. If I fail. I try again right away to do better.	2.67 (0.89)	2.75 (0.88)	2.29 (0.93)	2.29 (0.94)	2.50 (0.88)	2.47 (0.88)
3. There are things I really care about.	3.39 (0.80)	3.20 (0.95)	3.34 (0.86)	2.92 (1.08)	3.34 (0.91)	3.38 (0.80)
4. I notice my thoughts and feelings.	3.04 (0.89)	3.02 (0.88)	2.95 (0.89)	2.72 (0.96)	3.07 (0.89)	3.05 (0.80)
5. I give up when things are too hard.*	2.38 (1.03)	2.47 (1.06)	2.09 (1.14)	2.40 (0.93)	2.13 (1.05)	2.36 (0.99)
6. Nothing matters that much to me.*	3.32 (0.93)	3.18 (1.04)	3.16 (0.98)	3.08 (1.00)	3.26 (0.97)	3.27 (0.96)
7. It’s OK to be scared.	2.83 (1.12)	2.27 (1.30)	2.76 (1.11)	1.93 (1.35)	2.64 (1.08)	2.14 (1.18)
8. If I think something. that doesn’t mean it’s true.	2.48 (1.08)	2.63 (1.13)	2.60 (0.99)	2.45 (1.14)	2.55 (0.98)	2.60 (1.02)
9. Sometimes I don’t notice what’s happening or what people are saying.*	1.78 (1.02)	1.98 (1.13)	1.58 (1.02)	2.08 (1.10)	1.76 (1.15)	1.89 (1.06)
10. My thoughts don’t make me do what I do.	1.94 (0.94)	2.11 (1.14)	1.82 (1.03)	1.96 (1.02)	1.92 (1.02)	2.11 (1.00)
11. It’s OK to feel mad.	2.99 (1.00)	2.90 (1.07)	3.04 (0.92)	2.63 (1.06)	2.97 (0.97)	2.84 (0.95)
12. If I do something bad. then I’m a bad person.*	2.47 (1.12)	2.70 (1.14)	2.48 (1.16)	2.52 (1.10)	2.56 (1.14)	2.75 (1.10)
13. I worry a lot about stuff I did or need to do.*	1.55 (1.15)	2.20 (1.08)	1.39 (1.03)	2.12 (1.15)	1.17 (1.08)	1.86 (1.06)
14. I notice when my body feels different.	2.43 (1.05)	2.28 (1.19)	2.64 (1.02)	1.99 (1.17)	2.53 (0.97)	2.33 (1.15)
15. If I get angry. it means I messed up.*	2.42 (1.15)	2.71 (1.05)	2.26 (1.16)	2.83 (1.06)	2.40 (1.14)	2.82 (0.97)
16. My thoughts and feelings tell me what to do.*	1.85 (1.02)	2.06 (1.14)	1.69 (0.90)	2.10 (1.09)	1.70 (0.87)	1.93 (0.99)
17. I am what other people say about me.*	2.38 (1.16)	2.76 (1.15)	2.48 (1.07)	2.56 (1.06)	2.58 (1.03)	2.60 (1.08)
18. Grown-ups tell me what is important to me.*	2.21 (1.22)	2.11 (1.30)	2.64 (0.98)	2.45 (1.26)	2.64 (1.12)	2.46 (1.13)
Total Score	45.00 (6.77)	46.50 (7.09)	44.20 (6.83)	43.60 (5.98)	44.30 (1.12)	45.6 (6.77)

Note. * = reverse-scored items, ^a^ = M (SD)

#### Factor retention

**Fig. 1:**
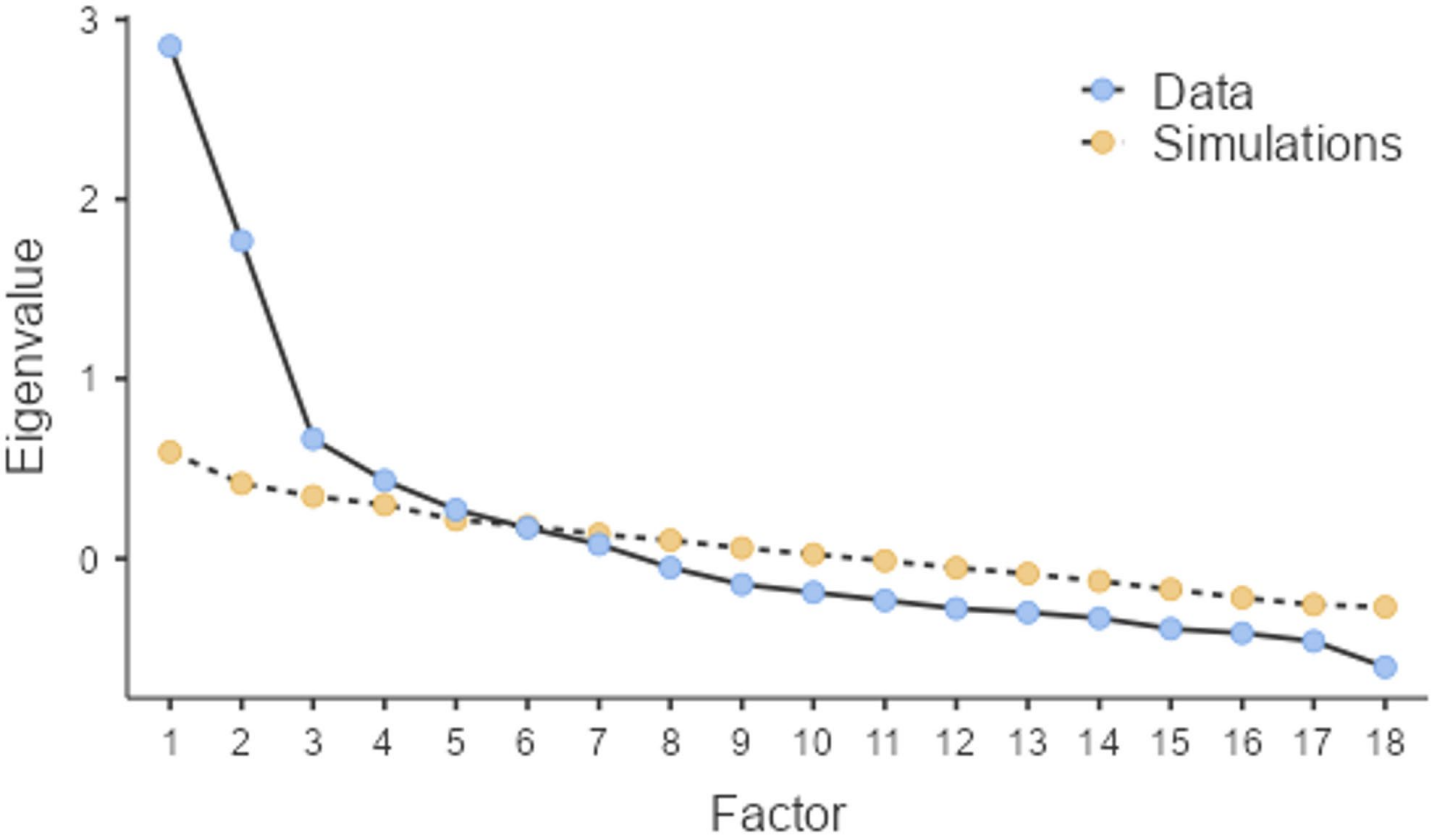
Parallel Analysis Scree Plot Note. Data = Initial Eigenvalues, Simulations = Simulated Eigenvalues

All three factor retention criteria indicated different numbers of factors. The MAP test indicated a 2-factor structure, while the scree plot indicated three factors and the parallel analysis five (see Fig. 1 for parallel analysis scree plot and Appendix C for exact initial and simulated eigenvalues). The two-factor solution appeared somewhat noisy, with numerous items showing problematically low communalities (11 items < 0.30), whereas the five-factor model included three two-item factors (primary loadings > 0.40), contributing with instability to the structure. Accordingly, we selected the three-factor model as the basis for the subsequent item-reduction process.

#### Item retention

The three-factor model explained 32.7% of the variance but displayed a somewhat messy structure, with several items showing cross-loadings and low communalities. The iterative item retention process proceeded in nine rounds, during which eight items were removed based on a combination of low primary loading and low communality, in the following order: 10, 16, 14, 9, 17, 18, 6, and 5 (see Table 3 for item content). In the final refinement round, item 3 was removed due to a cross-loading difference of less than 0.10 and poor conceptual fit with its primary factor, despite having a primary loading that approached 0.40 and adequate communality. See Appendix D for the output from each round of the item retention process.

The final nine-item model produced a clean and interpretable structure (Table 3), with each factor including three items and the model explaining 46.5% of the variance. The retained items met all inclusion criteria except for item 12, that had a communality (h^2^ = 0.27), slightly below the threshold. This item was retained because it met all criteria related to factor loadings (C1-C3), aligned theoretically with Factor 3, and contributed to maintaining a stable structure of three items per factor.

This shorter form was labeled CPFQ-9, including the following factors: FA (1) committed action with awareness (items 1, 2, and 4); FA (2) acceptance and defusion (items 7, 8, and 11), and FA (3) fusion and self-judgement (items 12, 13, and 15).

**Table 3:** Items and factor loadings for the CPFQ-9

CPFQ Item	FA 1	FA 2	FA 3	h^2^
1. I try really hard every day.	0.64			0.42
2. If I fail, I try again right away to do better.	0.86			0.72
4. I notice my thoughts and feelings.	0.48			0.38
7. It’s OK to be scared.		0.61		0.43
8. If I think something, that doesn’t mean it’s true.		0.52		0.37
11. It’s OK to feel mad.		0.75		0.53
12. If I do something bad, then I’m a bad person.			0.51	0.27
13. I worry a lot about stuff I did or need to do.			0.61	0.45
15. If I get angry, it means I messed up.			0.81	0.65

Note. FA 1 = Committed Action with Awareness; FA 2 = Acceptance and Defusion; FA 3 = Fusion and Self-Judgement; h^2^ = Communality

#### Inter-Factor correlations and internal consistency

Factors 1 and 2 were modestly correlated (*r* =.38), while correlations between Factor 1 and Factor 3 (*r* =.06), and Factor 2 and Factor 3 (*r* = −.09) were low, suggesting different constructs and supporting the use of oblique rotation. Reliability analyses showed acceptable or borderline internal consistency for the different factors: α = 0.71 (Factor 1), α = 0.68 (Factor 2), and α = 0.68 (Factor 3). The full scale showed questionable internal consistency (α = 0.60), indicating that interpretation is more appropriate at the subscale level. To complement alpha, we also computed McDonald’s ω total. The results were consistent with α, with ω being 0.73 for Factor 1, 0.68 for Factor 2, 0.69 for Factor 3, and 0.65 for the full scale.

### Confirmatory factor analysis

In the CFA sample, complete data on all 18 items were available for 781 participants (84.0%) and item-level missingness was low (3–25). Little’s MCAR test indicated that the data were consistent with MCAR (X^2^ [105] = 108.624, *p* =.385). All items showed skewness below 0.2 and kurtosis below 0.7, indicating no severe violations of univariate normality. However, Mardia’s tests for skewness and kurtosis were both significant (*p* <.001), suggesting deviations from multivariate normality and justifying the use of robust maximum likelihood estimation (MLR).

We proceeded to model the nine-item three-factor model, using the full CFA sample. All factor loadings were statistically significant, ranging from 0.42 to 0.78, with eight of the nine items loading above 0.50. The chi-square test indicated a statistically significant (*p <*.001) model misfit, and the TLI (0.882) was slightly below the cutoff for acceptable fit. However, RMSEA (0.069), SRMR (0.057), and CFI (0.921) were all within the acceptable range. Examination of the modification indices suggested that adding a residual covariance between items 1 and 2 would improve model fit substantially (MI = 27.8). As these items overlap in content and load on the same factor (Factor 1), allowing the residual correlation was theoretically justified.

**Fig. 2:**
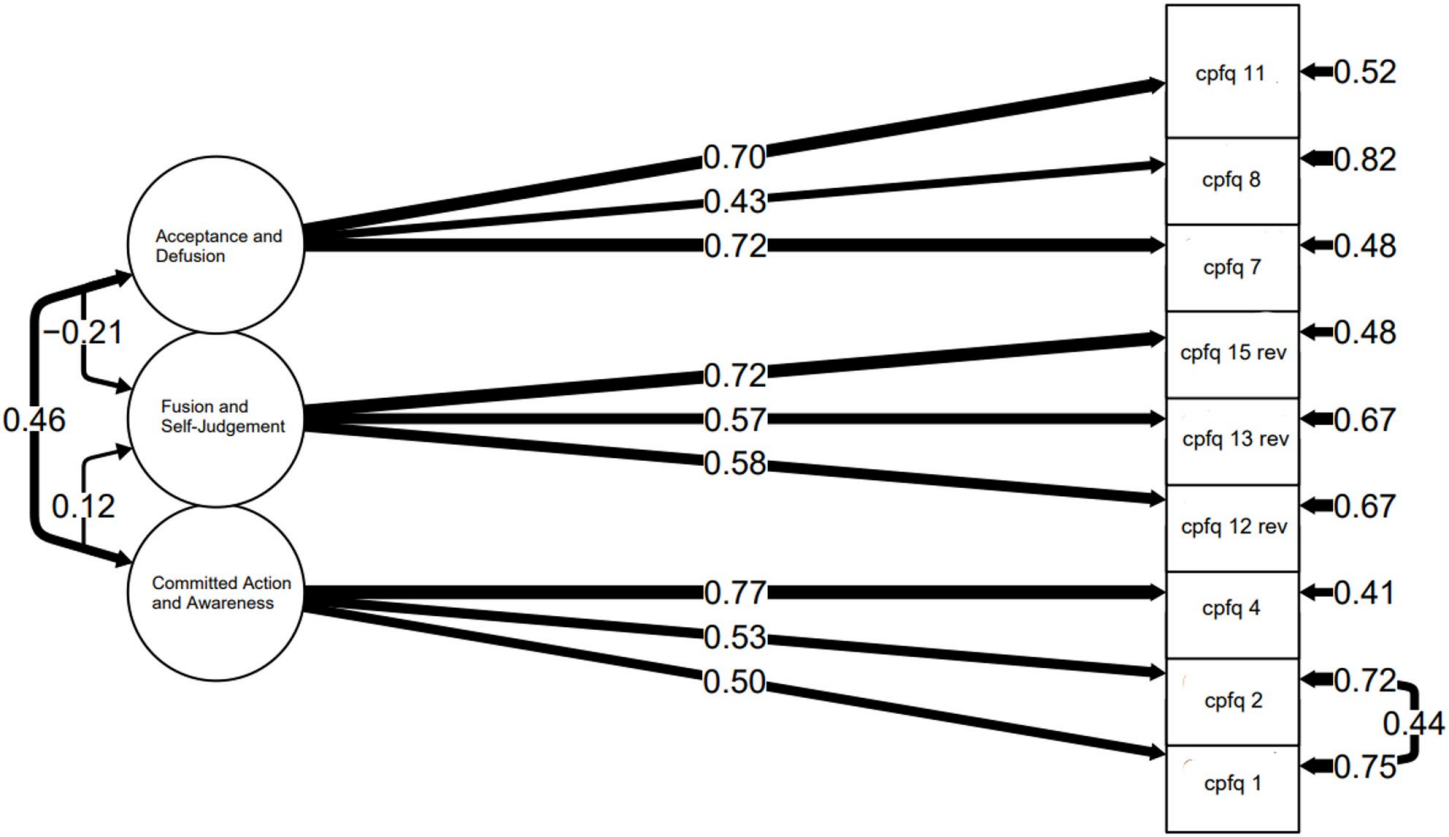
Confirmatory Factor Analysis Model of the CPFQ-9

A revised model was therefore tested with this residual covariance included. Although the Chi-Square test continued to be statistically significant, all other fit indices suggested acceptable model fit and a clear improvement compared to the more constrained model (see Table 4). All factor loadings remained statistically significant, ranging from 0.43 to 0.77 (see Fig. 2). The inter-factor correlations indicated a moderate positive correlation between Factors 1 and 2 (*r* =.46), a weak positive correlation between Factors 1 and 3 (*r* =.12), and a weak negative correlation between Factors 2 and 3 (*r* = −.21).

The average inter-item correlations were 0.45 for Factor 1, 0.37 for Factor 2, and 0.38 for Factor 3, indicating sufficient homogeneity without redundancy. Estimation of internal consistency using Cronbach’s alpha indicated borderline-to-acceptable reliability for Factor 1 (α = 0.71), Factor 2 (α = 0.63) and Factor 3 (α = 0.65). The omega total coefficients were 0.57 for Factor 1, 0.66 for Factor 2, and 0.66 for Factor 3. The lower omega value for Factor 1 reflects that omega accounts for the specified measurement model, including the residual covariance.

### Measurement invariance

Measurement invariance testing was conducted using multi-group CFAs across grade and gender at three levels: configural, metric, and scalar. Model fit indices for all models are presented in Table 4. The models assessing configural and metric invariance met the established criteria for both grade and gender. However, full scalar invariance was not supported in either grouping variable, indicating that some item intercepts were not equivalent across groups. To achieve partial scalar invariance, item intercept constraints were released sequentially, beginning with those that contributed most to model misfit. This procedure was continued until an acceptable level of partial invariance was obtained, ensuring that no more than one intercept per factor was freely estimated. For the grade variable, partial scalar invariance was achieved by freeing the intercept of item 4, whereas for the gender groups, the intercepts of items 2, 7, and 13 were freed.

**Table 4:** Measurement invariance model fit indices across grade and gender groups

Group	Model	X^2^	df	CFI	ΔCFI	TLI	ΔTLI	SRMR	RMSEA	ΔRMSEA	90% CI RMSEA
Total sample	Constrained model	128.480***	24	0.917		0.875		0.057	0.069		[0.058, 0.080]
	Adjusted model^a^	100.179***	23	0.939		0.904		0.051	0.060		[0.049, 0.072]
Grade	Configural	144.526***	69	0.939		0.904		0.055	0.060		[0.047, 0.072]
	Metric	159.474***	81	0.937	0.002	0.915	0.011	0.060	0.056	0.003	[0.044, 0.068]
	Scalar	211.544***	93	0.904	0.033	0.889	0.026	0.067	0.064	0.008	[0.053, 0.075]
	Partial scalar^b^	186.585***	91	0.923	0.014	0.908	0.007	0.063	0.058	0.002	[0.047, 0.070]
Gender	Configural	115.179***	46	0.944		0.912		0.053	0.057		[0.045, 0.069]
	Metric	131.752***	52	0.935	0.009	0.911	0.001	0.058	0.058	0.001	[0.046, 0.069]
	Scalar	213.864***	58	0.874	0.061	0.843	0.068	0.068	0.076	0.018	[0.066, 0.087]
	Partial scalar^c^	142.413***	55	0.929	0.055	0.907	0.064	0.060	0.059	0.017	[0.047, 0.070]

Note. X2 = Chi-Square Test, *df* = degrees of freedom, CFI = Comparative Fit Index, TLI = Tucker Lewis Index, SRMR = Standardized Root Mean Square Residuals, RMSEA = Root-Mean-Square Error of Approximation, CI = Confidence Interval

^a^Residuals of items 1 and 2 correlated

^b^Intercept of item 4 freed

^c^Intercepts of items 2, 7, and 13 freed

^d^*p <.05. **p <.01. ***p <.001

## Discussion

This study aimed to inform both theory and practice by investigating the structure of psychological flexibility (PF) and inflexibility (PI) in early- and mid-adolescence, and by providing a preliminary validation of a brief, multidimensional measure for use in a school setting, the CPFQ-9. Using a large sample spanning from early to mid – adolescence, this is, to our knowledge, the first structural validation of a multidimensional PF/PI– measure specifically developed for younger populations that employs both exploratory and confirmatory factor analyses, with tests of measurement invariance across relevant groups. Through an iterative, exploratory item-retention process, we identified a parsimonious 9-item, three-factor structure, which was replicated in a split sample and demonstrated configural, metric, and partial scalar invariance across different ages and gender.

### Theoretical considerations

The resulting structure consisted of two PF dimensions (Factor 1 and 2) and one PI dimension (Factor 3). Although the items are factors are derived from a PF/PI framework, the results can also be interpreted through a broader developmental lens, with conceptual parallels to other regulatory and vulnerability constructs. Specifically, the CPFQ-9 factors align conceptually with three widely recognized domains of functioning: behavioral regulation, emotional regulation, and negative affect.

Factor 1, labeled committed action with awareness, reflects a PF domain characterized by sustained engagement in goal-directed behavior, even in the face of difficulties or setbacks. Conceptually, this aligns with the “active” component of the triflex model [54, 55]. In contrast to related measures [7, 13], the items in this domain describe sustained persistence in goal-directed behaviors without incorporating the component of value congruence. This may reflect differences in item wording: in the CPFQ item set, committed action and values are represented by separate items, whereas other instruments combine these constructs within the same items [7, 13]. In our analyses, the “pure” values items – which describe insight into what is important to oneself – did not load on this domain of overt behavior. Instead, the factor suggests a functional connection between attentional awareness and committed action. As early and mid-adolescents are still in the process of clarifying their personal values, the attentional capacity for noticing may serve as a more potent resource for regulating goal-directed behavior, before the establishment of the reinforcing function of personally chosen values. The aspects of persistence and goal-directed behavior reflected in this factor correspond theoretically to developmentally relevant models of behavioral regulation. Such models include temperament theories of effortful control [56] as well as executive function frameworks that describe the gradual maturation of prefrontal cortical systems supporting sustained attention and flexible goal maintenance across adolescence [57]. The component of attentional awareness included in the factor aligns with literature indicating a link between mindfulness skills and behavioral regulation [58, 59], as reflected in the concept of “acting with awareness”, which is described as a core facet of mindfulness [5]. Considering these convergent theoretical aspects, the factor could be conceptualized through the lens of a broader attentional-behavioral regulatory system, rather than just the narrower PF/PI construct of committed action.

Factor 2, labeled acceptance and defusion, represents a PF domain characterized by a non-judgmental and flexible stance toward internal experiences. This factor is a clear reflection of the “open” dimension that has emerged in other multidimensional PF/PI measures [7, 13]. However, it differs from dimensions in related instruments in that it consists of positively worded items capturing the PF variant of the dimension, instead of negatively worded items that align with the PI processes of fusion and experiential avoidance. More broadly, this factor is consistent with developmental models of emotion regulation, which suggest that strategies such as acceptance, cognitive distancing and reappraisal become progressively more prominent as individuals move from childhood into adolescence - a typical developmental trajectory in which emotion regulation gradually transitions from reliance of external sources to the development of internally guided regulatory capacities [60, 61].

Factor 3, labeled fusion and self-judgment, represents a PI domain characterized by rigid entanglement with negative self-evaluations and a narrowing of attentional processes The factor appears to reflect a broad dimension of tendency for negative affect marked by worry, rumination, and self-criticism, aligning it with established vulnerability constructs such as neuroticism [62]. Interestingly, this factor emerged independently of the two PF, “regulatory” factors – a pattern consistent with evidence demonstrating the relative independence between flourishing and distress [63], as also reflected in the largely orthogonal relationship that has been demonstrated between PF and PI [12, 26].

These findings parallel the dual-factor model of mental health, which conceptualizes well-being and distress as partially independent dimensions [64]. PF-related factors may capture self-regulatory skills that operate as upstream mechanisms, helping explain why flourishing and distress can cooccur (e.g., “symptomatic-but-content” profiles). Such a model underscores the importance of monitoring PF and PI processes in parallel during screening and intervention evaluation, given that strategies that influence one (e.g. PF) do not necessarily influence the other (e.g. PI).

Moreover, regulatory dimensions such as Factors 1 and 2 may represent more meaningful operationalizations of intervention focus in preventive contexts, where evaluations are often constrained by floor effects on symptom measures [65]. From both developmental and intervention-science perspectives, an interesting future direction for research is to examine interactions between the two PF factors and the PI factor as facilitators for intervention progress. For example, whether the developmental trajectories of these three systems moderate the effectiveness of PF/PI interventions. A related question of interest is whether more advanced behavioral and emotional regulation capacities serve a scaffolding function when learning ACT principles, or whether heightened vulnerability to negative affect functions as a barrier to such development.

### Developmental considerations

As noted by Petersen et al. (2024) [3] there is a need for empirical validation of the hexaflex model in younger populations, as PF/PI subprocesses may not be as discretely separated as the model suggests. Our study is in line with a growing body of research [7, 12, 13] suggesting that PF/PI processes may be better represented by more parsimonious models. This is not necessarily only a statistical artefact, but may reflect the developmental trajectory of PF/PI processes. As others have pointed out, it is likely that not all PF/PI processes are equally salient at all stages of development [3], and that some processes only become fully available at later stage of maturation. For example, the perspective-taking capacity described in the self-as-context process, or a non-literal, defused stance toward thoughts, requires well-developed metacognitive and linguistic resources, whereas the concept of personally chosen values may become more strongly and contingently linked to overt behavior towards the end of adolescence, when identity formation and value clarification become central developmental tasks.

On the other hand, unidimensional measures of only PF or only PI appear as too narrow to represent the broader PF/PI constructs. Interventions for children and adolescents may therefore benefit from targeting a smaller number of functional clusters, rather than being structured around each separate hexaflex process [3, 66]. For example, PF/PI intervention procedures may be most effective when they are explicitly targeted toward the three dimensions found in these results. Committed action and present-moment awareness may support the development of behavioral regulation, acceptance and defusion techniques may strengthen the “emotion regulation system”, and targeting fusion, rigid self-judgment, and inattention may help reduce vulnerability to negative affect. This dual-factor perspective where PF and PI represent concurrent constructs of both protection and vulnerability, also highlights the importance of multidimensional assessment within youth-focused PF/PI interventions. The use of multidimensional measures in youth intervention studies is currently strikingly rare and the vast majority of PF/PI evidence in intervention studies is still relying on the unidimensional AFQ (blinded reference, manuscript in preparation).

### Preliminary validation of the CPFQ-9: contributions and remaining questions

#### Structural validity and measurement invariance

This study provides structural evidence for the CPFQ-9 as a brief and multidimensional PF/PI measure that is designed specifically for children and adolescents. Its brevity makes it suitable for routine use in time-constrained environments such as school settings. The factor structure was first identified using established criteria in EFA and then replicated with CFA across ages ranging from early to mid-adolescence. Structural validity is further supported by evidence of configural, metric, and partial scalar invariance across grade levels and gender.

Given the partial scalar invariance demonstrated in the present study, the comparison of latent means between genders and grade levels is warranted using the less constrained model. However, such comparisons should be interpreted with appropriate caution until additional evidence is available regarding construct validity. This includes convergent validity with established PF/PI measures and divergent validity with theoretically related but distinct constructs. Furthermore, evidence regarding the CPFQ-9’s concurrent and predictive validity is needed to clarify how the subscales are associated with relevant PF/PI-related outcomes, such as symptomatology and indicators of positive well-being.

It should also be noted that although the CPFQ items are intended to be “easy to understand” and “child-friendly” [30] and suitable for different age groups, the validity of this measure would benefit from assessing the comprehensibility of the individual items. Such refinement of the item content and wording could, for example, be carried out through cognitive interviews, similar to the procedure adopted during the development of CompACT-Y [13].

#### Reliability

Estimates of internal consistency for the subscales indicated questionable to borderline acceptable reliability. The most notable concern was observed for Factor 1, whose omega value declined from 0.73 to 0.57 when the residual covariance between Items 1 and 2 was included in the CFA model. Low internal consistency is, however, common for short subscales [46, 67, 68]. Some scholars therefore recommend examining average inter-item correlations as an index of within-subscale homogeneity and redundancy [46]. On this metric, all CPFQ-9 subscales, including Factor 1, fell within the acceptable range of 0.15–0.50.15.50. Nevertheless, the current evidence suggests that the CPFQ-9 is not necessarily sufficiently reliable for making inferences at the individual level. Rather, it appears more appropriate for research purposes or for use at the group level. Further evidence is also required regarding other forms of reliability, including test-retest reliability, and sensitivity for change in intervention contexts.

#### Contributions and advantages of the CPFQ-9

Although the CPFQ-9 shows conceptual and structural overlap with related measures, particularly the CompACT-Y, it offers several contributions that may advance the youth PF/PI literature. First, as stated, this is, to the best of our knowledge, the first multidimensional PF/PI instruments for younger populations whose structure has been validated using both EFA and CFA, as well as measurement invariance testing across age and gender. Second, its short length represents a distinctive strength, as existing multidimensional measures are longer and therefore less feasible in applied or intervention contexts where time is limited. Third, our sample had a younger mean age (6th graders majority in the CFA subsample) than the validation sample of the CompACT-y [13]. Our items were adapted from a parent instrument whose appropriateness has been demonstrated in even younger children. This provides a promising basis for the CPFQ-9 to be a suitable measure for younger age groups as well. However, the structural validity of the CPFQ-9 requires replication in samples that include younger children in order to provide evidence for the developmental generalizability of the measure. Lastly, the CPFQ-9 comprises two factors consisting of positively worded items that capture the *presence* of strategies that are in line with two core developmental regulatory systems (behavioral and emotional) and a third factor that reflects a general tendency toward worry and negative affectivity. This broader coverage of adaptive behaviors may therefore make it even more relevant for use in universal interventions, where the primary focus is on the strengthening of adaptive functioning rather than the reduction of symptomatology.

#### Contextual considerations

Contextual factors need to be accounted for when interpretating the validity evidence. Since the scale was administered right before a mathematics assessment, the resulting factor structure may reflect context-specific self-evaluations rather than more global PF/PI responses. For example, Factor 1 includes two items related to persistence under difficulty, which could be considered a self-regulatory stance likely influenced by the exam setting and participants’ beliefs about their academic abilities. Cross-cultural and contextual validation is required to generalize finding beyond the current sample. Also, translation effects and comprehension difficulties may influence responses, and should be evaluated before to generalizing to linguistic groups other than Finnish-speaking students.

#### Implications for the original CPFQ

Lastly, although the CPFQ-9’s items were based on the original 24-item CPFQ, these results should not be considered as direct validation evidence for the 24-item version. We used only a subset of items, reworded several items, and altered the order of presentation; all of which can affect the structure of an item set. Future validation work on the full 24-item CPFQ may still benefit the continued development of the CPFQ-9. For example, some of the six excluded items that we excluded from the 24-item set may improve our model, such as the item “I know what I want to work for today”, which could shed further light on the relationships between behavioral persistence, value clarification, and present-moment awareness.

## Conclusions

The present study contributed to the literature on the structure of psychological flexibility and inflexibility (PF/PI) in early and mid-adolescence by providing structural evidence for a brief, multidimensional questionnaire with potential applicability in time-constrained environments, such as the school context. Our model included two PF dimensions, committed action with awareness and defusion and acceptance, reflecting mindful and persistent engagement in goal-directed behavior and an open and non-judgmental stance towards internal experiences. The single PI dimension, labeled fusion and self-judgment, reflected narrowing of attention and entanglement with negative self-concepts. This structure corresponds to two developmentally relevant regulatory systems – behavioral and emotional regulation – as well as one vulnerability factor, conceptually aligned with constructs of negative affectivity. The structure parallels the dual-factor model of mental health, in which adaptive functioning and distress are regarded as independent dimensions.

The structural validation of the CPFQ-9 provides a promising foundation for brief multidimensional PF/PI assessment in youth. However, further work is needed regarding validity, reliability, and generalization to earlier developmental stages before the instrument can be considered a genuinely developmentally sensitive, and ready-for-use measure.

## Supplementary Information

Supplementary Material 1.

## Data Availability

The data supporting our findings in this study are not publicly available due to data privacy laws protecting the personal information of the participants. However, anonymized data can be obtained upon reasonable request from Katarina Alanko ([katarina.alanko@utu.fi](https:/utufi-my.sharepoint.com/personal/jblang_utu_fi/Documents/katarina.alanko@utu.fi)).The 18-item Finnish version of the Child Psychological Flexibility Questionnaire (CPFQ) is available upon request from the corresponding author ([jakob.b.langenskiold@utu.fi](mailto: jakob.b.langenskiold@utu.fi)).

## Abbreviations

AAQ-II: Acceptance and Action Questionnaire–II
ACT: Acceptance and Commitment Therapy
AFQ-Y: Avoidance and Fusion Questionnaire for Youth
AIIC: Average Inter-Item Correlation
CFA: Confirmatory Factor Analysis
CFI: Comparative Fit Index
CI: Confidence Interval
CompACT-Y: Comprehensive Assessment of Acceptance and Commitment Therapy – Youth
CPFQ: Children’s Psychological Flexibility Questionnaire
d: Cohen’s d
df: Degrees of freedom
EFA: Exploratory Factor Analysis
FA: Factor
KMO: Keiser–Meyer–Olkin (measure of sampling adequacy)
M: Mean
MAP: Minimum Average Partial
MCAR: Missing Completely at Random
MI: Measurement Invariance
N: Sample size
p: *p*-value
PAF: Principal Axis Factoring
PF/PI: Psychological Flexibility / Psychological Inflexibility
RMSEA: Root Mean Square Error of Approximation
SD: Standard Deviation
SRMR: Standardized Root Mean Square Residual
TLI: Tucker-Lewis Index
α: Cronbach’s alpha
ω: McDonald’s omega
