# Supplementary material for: Structural validation of a brief, multidimensional measure of psychological flexibility and inflexibility in adolescence
Source: BMC Psychol. 2026 Jan 10;14:192. doi: 10.1186/s40359-025-03937-w (PMC12882279; doi:10.1186/s40359-025-03937-w)
Supplement: Supplementary file 1 — Supplementary Material 1. [file 40359_2025_3937_MOESM1_ESM.docx]

**Appendix A**

**The Original 24-Item Children’s Psychological Flexibility Questionnaire**

| **Table A1** |  |  |  |
| --- | --- | --- | --- |
| *Original 24-Item CPFQ Item Set and Inclusion in the Shorter Version* | | |  |
|  |  |  |  |
| Item | Hypothesized Process | Included in 18-Item Version |  |
| 1 I always notice things around me and what people say. | Present Moment | No |  |
| 2 If I think something, that doesn't mean it's true. | Defusion | Yes |  |
| 3 There are things that I really care about. | Values | Yes |  |
| 4 If I do something bad, then I am a bad person. | Self-As-Context | Yes |  |
| 5 I notice when my body feels different. | Present moment | Yes |  |
| 6 When I mess up, I get mad at myself. | Committed Action | No |  |
| 7 Nothing matters that much to me. | Values | Yes |  |
| 8 If I cry it means that I'm wrong or bad. | Acceptance | No |  |
| 9 It's OK to be scared. | Acceptance | Yes |  |
| 10 I notice my thoughts and feelings, but that is not me. | Self-As-Context | Yes |  |
| 11 I miss seeing stuff happen or hearing what people say. | Present Moment | Yes |  |
| 12 My thoughts don't make me do what I do. | Defusion | Yes |  |
| 13 Everything I think and feel must be real. | Defusion | No |  |
| 14 It's OK to feel mad. | Acceptance | Yes |  |
| 15 I know what I want to work for today. | Values | No |  |
| 16 If I lose, I try again right away to do better. | Committed Action | Yes |  |
| 17 I give up when things are too hard. | Committed Action | Yes |  |
| 18 I worry a lot about stuff I did or need to do. | Present Moment | Yes |  |
| 19 If I get angry, it means I messed up. | Acceptance | Yes |  |
| 20 My thoughts and feelings tell me what to do. | Defusion | Yes |  |
| 21 I am what other people say about me. | Self-As-Context | Yes |  |
| 22 If I did something wrong, that doesn't make me bad. | Self-As-Context | No |  |
| 23 Grown-ups tell me what is important to me. | Values | Yes |  |
| 24 I try really hard every day. | Committed Action | Yes |  |

**Appendix B**

**Forward-Back Translation of the 18-Items**

| **Table B1** |  |
| --- | --- |
| *Forward-Back Translations of the 18-item Set* |  |
|  |  |
| Item | Version |
| 1. I try really hard every day. | Original Item |
| 1. Yritän parhaani joka päivä. | Forward (Finnish) |
| 1. I try my best every day. | Back (English) |
| 2. If I fail. I try again right away to do better. | Original Item |
| 2. Kun epäonnistun, yritän uudestaan heti, jotta voisin onnistua paremmin. | Forward (Finnish) |
| 2. When I fail, I try again right away in order to do better. | Back (English) |
| 3. There are things I really care about. | Original Item |
| 3. On asioita, joista todella välitän. | Forward (Finnish) |
| 3. There are things that really matter to me. | Back (English) |
| 4. I notice my thoughts and feelings. | Original Item |
| 4. Huomaan, mitä ajattelen ja tunnen. | Forward (Finnish) |
| 4. I notice what I think and feel. | Back (English) |
| 5. I give up when things are too hard. | Original Item |
| 5. Luovutan, kun asiat ovat liian vaikeita. | Forward (Finnish) |
| 5. If things are too difficult, I give up. | Back (English) |
| 6. Nothing matters that much to me. | Original Item |
| 6. Mikään ei tunnu tärkeältä minulle. | Forward (Finnish) |
| 6. Nothing feels important to me. | Back (English) |
| 7. It's OK to be scared. | Original Item |
| 7. On ihan ok olla peloissaan. | Forward (Finnish) |
| 7. It’s ok to feel scared. | Back (English) |
| 8. If I think something. that doesn’t mean it’s true. | Original Item |
| 8. Jos ajattelen jotain, se ei tarkoita, että se on totta. | Forward (Finnish) |
| 8. If I think of something, it doesn’t mean it’s true. | Back (English) |
| 9. Sometimes I don’t notice what’s happening or what people are saying. | Original Item |
| 9. Joskus en huomaa, mitä ympärilläni tapahtuu tai mitä ihmiset sanovat. | Forward (Finnish) |
| 9. Sometimes I don’t notice what’s happening around me or what people say. | Back (English) |
| 10. My thoughts don’t make me do what I do. | Original Item |
| 10. Ajatukseni eivät määrää, mitä teen. | Forward (Finnish) |
| 10. My thoughts don’t determine me what I do. | Back (English) |
| 11. It’s OK to feel mad. | Original Item |
| 11. On ihan ok tuntea että on vihainen. | Forward (Finnish) |
| 11. It’s ok to feel angry. | Back (English) |
| 12. If I do something bad. then I’m a bad person. | Original Item |
| 12. Jos teen jotain pahaa, silloin olen paha ihminen. | Forward (Finnish) |
| 12. If I do something bad, it means I’m a bad person. | Back (English) |
| 13. I worry a lot about stuff I did or need to do. | Original Item |
| 13. Huolestun usein asioista, joita olen tehnyt tai joita minun täytyy tehdä. | Forward (Finnish) |
| 13. I often get worried about things that I have done of that I have to do. | Back (English) |
| 14. I notice when my body feels different. | Original Item |
| 14. Huomaan, kun kehoni tuntuu erilaiselta. | Forward (Finnish) |
| 14. I notice when my body feels different. | Back (English) |
| 15. If I get angry. it means I messed up. | Original Item |
| 15. Jos suutun, se tarkoittaa että pilasin asiat. | Forward (Finnish) |
| 15. If I get angry, it means that I screwed up. | Back (English) |
| 16. My thoughts and feelings tell me what to do. | Original Item |
| 16. Ajatukseni ja tunteeni kertovat minulle mitä minun tulee tehdä. | Forward (Finnish) |
| 16. My thoughts and feelings tell me what I should do. | Back (English) |
| 17. I am what other people say about me. | Original Item |
| 17. Olen sellainen, mitä muut sanovat minusta. | Forward (Finnish) |
| 17. I am that kind of person what other people say about me. | Back (English) |
| 18. Grown-ups tell me what is important to me. | Original Item |
| 18. Aikuiset kertovat minulle, mikä on minulle tärkeää. | Forward (Finnish) |
| 18. Adults tell me what is important. | Back (English) |

**Appendix C**

**Eigenvalues From the Exploratory Factor Analysis**

| **Table C1** |  |  |  |  |  |  |  |
| --- | --- | --- | --- | --- | --- | --- | --- |
| *Observed and Simulated Eigenvalues for the 18-item Set* | | | | | | |  |
|  |  |  |  |  |  |  |  |
| Factor | Observed | Simulated |  |  |  |  |  |
| 1 | 2.739 | 0.518 |  |  |  |  |  |
| 2 | 1.824 | 0.364 |  |  |  |  |  |
| 3 | .692 | .288 |  |  |  |  |  |
| 4 | .409 | .239 |  |  |  |  |  |
| 5 | .248 | .195 |  |  |  |  |  |
| 6 | .151 | .147 |  |  |  |  |  |
| 7 | .063 | .113 |  |  |  |  |  |
| 8 | -.036 | .068 |  |  |  |  |  |
| 9 | -.127 | .028 |  |  |  |  |  |
| 10 | -.209 | -.013 |  |  |  |  |  |
| 11 | -.241 | -.045 |  |  |  |  |  |
| 12 | -.270 | -.089 |  |  |  |  |  |
| 13 | -.296 | -.117 |  |  |  |  |  |
| 14 | -.331 | -.151 |  |  |  |  |  |
| 15 | -.386 | -.190 |  |  |  |  |  |
| 16 | -.423 | -.235 |  |  |  |  |  |
| 17 | -.469 | -.277 |  |  |  |  |  |
| 18 | -.600 | -.323 |  |  |  |  |  |

**Appendix D**

**Exploratory Factor Analyses and Item Reduction**

| **Table D1** | |  |  |  |  | |  |  | |  | |  | |
| --- | --- | --- | --- | --- | --- | --- | --- | --- | --- | --- | --- | --- | --- |
| *Round 1 of Item Reduction: Removal of Item 10* | |  |  |  |  | |  |  | |  | |  | |
|  |  |  |  |  |  | |  |  | |  | |  | |
| Items | | FA 1 | FA 2 | FA 3 | h^2^ | Decision | | | Flagging reason | | Reason for deletion | |  |
| 1. I try really hard every day. | | .48 | .10 | .04 | .27 | Retained* | | | C4 | |  | |  |
| 2. If I fail. I try again right away to do better. | | .68 | .05 | .09 | .50 | Retained | | |  | |  | |  |
| 3. There are things I really care about. | | .32 | .45 | .07 | .39 | Retained* | | | C2, C3 | |  | |  |
| 4. I notice my thoughts and feelings. | | .58 | .26 | .08 | .50 | Retained | | |  | |  | |  |
| 5. I give up when things are too hard. | | .37 | -.18 | .41 | .34 | Retained* | | | C2, C3 | |  | |  |
| 6. Nothing matters that much to me. | | .26 | .18 | .40 | .29 | Retained* | | | C3, C4 | |  | |  |
| 7. It's OK to be scared. | | .10 | .51 | -.13 | .33 | Retained | | |  | |  | |  |
| 8. If I think something. that doesn’t mean it’s true. | | .39 | .32 | -.11 | .34 | Retained* | | | C1, C2, C3 | |  | |  |
| 9. Sometimes I don’t notice what’s happening or what people are saying. | | .17 | -.32 | .31 | .22 | Retained* | | | C1, C2, C3, C4 | |  | |  |
| 10. My thoughts don’t make me do what I do. | | .23 | .01 | -.04 | .06 | Deleted | | | C1, C3, C4 | | Low loading, low h^2^ | |  |
| 11. It’s OK to feel mad. | | .09 | .66 | .04 | .48 | Retained | | |  | |  | |  |
| 12. If I do something bad. then I’m a bad person. | | -.05 | .13 | .54 | .29 | Retained* | | | C4 | |  | |  |
| 13. I worry a lot about stuff I did or need to do. | | .07 | -.36 | .50 | .41 | Retained* | | | C2, C3 | |  | |  |
| 14. I notice when my body feels different. | | .28 | .24 | -.29 | .26 | Retained* | | | C1, C3, C4 | |  | |  |
| 15. If I get angry. it means I messed up. | | .04 | -.01 | .72 | .52 | Retained | | |  | |  | |  |
| 16. My thoughts and feelings tell me what to do. | | -.18 | -.25 | .28 | .20 | Retained* | | | C1, C3, C4 | |  | |  |
| 17. I am what other people say about me. | | -.32 | .11 | .33 | .18 | Retained* | | | C1, C2, C3, C4 | |  | |  |
| 18. Grown-ups tell me what is important to me. | | -.46 | .29 | .38 | .32 | Retained* | | | C2, C3 | |  | |  |
|  | *Note*. * = Flagged item, C1 = primary loading <.40, C2 = secondary loading >.30, C3 = loading difference <.20, C4 = communality <.30 | | | | | | | | | | | | |

| **Table D2** | |  |  |  |  |  |  | |  | |  | |  | |
| --- | --- | --- | --- | --- | --- | --- | --- | --- | --- | --- | --- | --- | --- | --- |
| *Round 2 of Item Reduction: Removal of Item 16* | |  |  |  |  |  |  | |  | |  | |  | |
|  |  |  |  |  |  |  |  | |  | |  | |  | |
| Items | |  | FA 1 | FA 2 | FA 3 | h^2^ | | Decision | | Flagging reason | | Reason for deletion | |  |
| 1. I try really hard every day. | |  | .53 | .05 | -.01 | .29 | | Retained* | | C4 | |  | |  |
| 2. If I fail. I try again right away to do better. | |  | .71 | .00 | .05 | .52 | | Retained | |  | |  | |  |
| 3. There are things I really care about. | |  | .34 | .42 | .06 | .38 | | Retained* | | C2, C3 | |  | |  |
| 4. I notice my thoughts and feelings. | |  | .63 | .21 | .04 | .52 | | Retained | |  | |  | |  |
| 5. I give up when things are too hard. | |  | .37 | -.19 | .38 | .32 | | Retained* | | C1, C2, C3 | |  | |  |
| 6. Nothing matters that much to me. | |  | .30 | .15 | .38 | .29 | | Retained* | | C1, C2, C3, C4 | |  | |  |
| 7. It's OK to be scared. | |  | .10 | .51 | -.12 | .32 | | Retained | |  | |  | |  |
| 8. If I think something. that doesn’t mean it’s true. | |  | .35 | .33 | -.10 | .32 | | Retained* | | C1, C2, C3 | |  | |  |
| 9. Sometimes I don’t notice what’s happening or what people are saying. | | | .19 | .29 | -.33 | .29 | | Retained* | | C1, C3, C4 | |  | |  |
| 11. It’s OK to feel mad. | |  | .07 | .70 | .07 | .52 | | Retained | |  | |  | |  |
| 12. If I do something bad. then I’m a bad person. | |  | -.03 | .13 | .55 | .30 | | Retained | |  | |  | |  |
| 13. I worry a lot about stuff I did or need to do. | |  | .07 | -.34 | .49 | .40 | | Retained* | | C2, C3 | |  | |  |
| 14. I notice when my body feels different. | |  | .27 | .23 | -.30 | .26 | | Retained* | | C1, C3, C4 | |  | |  |
| 15. If I get angry. it means I messed up. | |  | .04 | .01 | .73 | .54 | | Retained | |  | |  | |  |
| 16. My thoughts and feelings tell me what to do. | |  | -.17 | -.25 | .27 | .19 | | Deleted | | C1, C3, C4 | | Low loading, low h^2^ | |  |
| 17. I am what other people say about me. | |  | -.28 | .09 | .34 | .16 | | Retained* | | C1, C3, C4 | |  | |  |
| 18. Grown-ups tell me what is important to me. | |  | -.40 | .25 | .38 | .26 | | Retained* | | C1, C2, C3, C4 | |  | |  |
|  | *Note*. * = Flagged item, C1 = primary loading <.40, C2 = secondary loading >.30, C3 = loading difference <.20, C4 = communality <.30 | | | | | | | | | | | |  | |

| **Table D3** | |  |  |  |  |  |  | |  | |  | |  | |
| --- | --- | --- | --- | --- | --- | --- | --- | --- | --- | --- | --- | --- | --- | --- |
| *Round 3 of Item Reduction: Removal of Item 14* | |  |  |  |  |  |  | |  | |  | |  | |
|  |  |  |  |  |  |  |  | |  | |  | |  | |
| Items | |  | FA 1 | FA 2 | FA 3 | h^2^ | | Decision | | Flagging reason | | Reason to delete | |  |
| 1. I try really hard every day. | |  | .55 | .06 | -.03 | .32 | | Retained | |  | |  | |  |
| 2. If I fail. I try again right away to do better. | |  | .73 | .00 | .03 | .54 | | Retained | |  | |  | |  |
| 3. There are things I really care about. | |  | .34 | .43 | .06 | .39 | | Retained* | | C2, C3 | |  | |  |
| 4. I notice my thoughts and feelings. | |  | .61 | .21 | .05 | .50 | | Retained | |  | |  | |  |
| 5. I give up when things are too hard. | |  | .37 | -.20 | .38 | .32 | | Retained* | | C1, C2, C3 | |  | |  |
| 6. Nothing matters that much to me. | |  | .29 | .14 | .38 | .29 | | Retained* | | C1, C3, C4 | |  | |  |
| 7. It's OK to be scared. | |  | .12 | .50 | -.12 | .33 | | Retained | |  | |  | |  |
| 8. If I think something. that doesn’t mean it’s true. | |  | .35 | .33 | -.09 | .31 | | Retained* | | C1, C2, C3 | |  | |  |
| 9. Sometimes I don’t notice what’s happening or what people are saying. | |  | .17 | -.35 | .30 | .24 | | Retained* | | C1, C2, C3, C4 | |  | |  |
| 11. It’s OK to feel mad. | |  | .07 | .69 | .06 | .51 | | Retained | |  | |  | |  |
| 12. If I do something bad. then I’m a bad person. | |  | -.03 | .13 | .53 | .28 | | Retained* | | C4 | |  | |  |
| 13. I worry a lot about stuff I did or need to do. | |  | .05 | -.33 | .51 | .41 | | Retained* | | C2, C3 | |  | |  |
| 14. I notice when my body feels different. | |  | .26 | .21 | -.28 | .22 | | Deleted | | C1, C3, C4 | | Low loading, low h^2^ | |  |
| 15. If I get angry. it means I messed up. | |  | .01 | .01 | .76 | .58 | | Retained | |  | |  | |  |
| 17. I am what other people say about me. | |  | -.28 | .08 | .33 | .16 | | Retained* | | C1, C3, C4 | |  | |  |
| 18. Grown-ups tell me what is important to me. | |  | -.39 | .25 | .37 | .24 | | Retained* | | C1, C2, C3, C4 | |  | |  |
|  | *Note*. * = Retained* item, C1 = primary loading <.40, C2 = secondary loading >.30, C3 = loading difference <.20, C4 = communality <.30 | | | | | | | | | | | |  | |

| **Table D4** | |  |  |  | |  | | |  |  | |  | |  | |
| --- | --- | --- | --- | --- | --- | --- | --- | --- | --- | --- | --- | --- | --- | --- | --- |
| *Round 4 of Item Reduction: Removal of Item 9* | |  |  |  | |  | | |  |  | |  | |  | |
|  |  |  |  |  | |  | | |  |  | |  | |  | |
| Items | | FA 1 | FA 2 | | FA 3 | | h^2^ | Decision | | | Flagging reason | | Reason to delete | |  |
| 1. I try really hard every day. | | .54 | .08 | | -.03 | | .32 | Retained | | |  | |  | |  |
| 2. If I fail. I try again right away to do better. | | .71 | .02 | | .04 | | .53 | Retained* | | |  | |  | |  |
| 3. There are things I really care about. | | .34 | .45 | | .06 | | .40 | Retained* | | | C2, C3 | |  | |  |
| 4. I notice my thoughts and feelings. | | .60 | .22 | | .06 | | .50 | Retained | | |  | |  | |  |
| 5. I give up when things are too hard. | | .36 | -.20 | | .38 | | .33 | Retained* | | | C1, C2, C3 | |  | |  |
| 6. Nothing matters that much to me. | | .28 | .14 | | .39 | | .29 | Retained* | | | C1, C3, C4 | |  | |  |
| 7. It's OK to be scared. | | .14 | .52 | | -.13 | | .35 | Retained | | |  | |  | |  |
| 8. If I think something. that doesn’t mean it’s true. | | .36 | .33 | | -.09 | | .30 | Retained* | | | C1, C2, C3 | |  | |  |
| 9. Sometimes I don’t notice what’s happening or what people are saying. | | .18 | -.35 | | .30 | | .25 | Deleted | | | C1, C3, C4 | | Low loading, low h^2^ | |  |
| 11. It’s OK to feel mad. | | .09 | .66 | | .07 | | .47 | Retained | | |  | |  | |  |
| 12. If I do something bad. then I’m a bad person. | | -.03 | .12 | | .53 | | .28 | Retained* | | | C4 | |  | |  |
| 13. I worry a lot about stuff I did or need to do. | | .07 | -.35 | | .49 | | .39 | Retained* | | | C2, C3 | |  | |  |
| 15. If I get angry. it means I messed up. | | .01 | -.01 | | .73 | | .54 | Retained | | |  | |  | |  |
| 17. I am what other people say about me. | | -.32 | .08 | | .36 | | .19 | Retained* | | | C1, C2, C3, C4 | |  | |  |
| 18. Grown-ups tell me what is important to me. | | -.41 | .25 | | .38 | | .26 | Retained* | | | C2, C3, C4 | |  | |  |
|  | *Note*. * = Flagged item, C1 = primary loading <.40, C2 = secondary loading >.30, C3 = loading difference <.20, C4 = communality <.30 | | | | | | | | | | | | | | |

| **Table D5** | |  |  |  |  |  | |  | |  |  | |  | |
| --- | --- | --- | --- | --- | --- | --- | --- | --- | --- | --- | --- | --- | --- | --- |
| *Round 5 of Item Reduction: Removal of Item 17* | |  |  |  |  |  | |  | |  |  | |  | |
|  |  |  |  |  |  |  | |  | |  |  | |  | |
| Items | |  | FA 1 | FA 2 | FA 3 | h^2^ | Decision | | Flagging reason | | | Reason to delete | |  |
| 1. I try really hard every day. | |  | .56 | .06 | -.03 | 0.33 | Retained | |  | | |  | |  |
| 2. If I fail. I try again right away to do better. | |  | .72 | .01 | .05 | 0.54 | Retained | |  | | |  | |  |
| 3. There are things I really care about. | |  | .35 | .40 | .06 | 0.38 | Retained* | | C1, C2, C3 | | |  | |  |
| 4. I notice my thoughts and feelings. | |  | .59 | .22 | .06 | 0.49 | Retained | |  | | |  | |  |
| 5. I give up when things are too hard. | |  | .36 | -.18 | .38 | 0.31 | Retained* | | C1, C2, C3 | | |  | |  |
| 6. Nothing matters that much to me. | |  | .28 | .14 | .38 | 0.28 | Retained* | | C1, C3, C4 | | |  | |  |
| 7. It's OK to be scared. | |  | .09 | .57 | -.15 | 0.39 | Retained | |  | | |  | |  |
| 8. If I think something. that doesn’t mean it’s true. | |  | .32 | .36 | -.10 | 0.31 | Retained* | | C1, C2, C3 | | |  | |  |
| 11. It’s OK to feel mad. | |  | .03 | .70 | .06 | 0.51 | Retained | |  | | |  | |  |
| 12. If I do something bad. then I’m a bad person. | |  | -.02 | .10 | .54 | 0.29 | Retained* | | C4 | | |  | |  |
| 13. I worry a lot about stuff I did or need to do. | |  | .07 | -.32 | .49 | 0.35 | Retained* | | C2, C3 | | |  | |  |
| 15. If I get angry. it means I messed up. | |  | .02 | -.01 | .76 | 0.58 | Retained | |  | | |  | |  |
| 17. I am what other people say about me. | |  | -.30 | .06 | .34 | 0.17 | Deleted | | C1, C2, C3, C4 | | | Low loading, low h^2^ | |  |
| 18. Grown-ups tell me what is important to me. | |  | -.41 | .24 | .36 | 0.25 | Retained* | | C2, C3, C4 | | |  | |  |
|  | *Note*. * = Flagged item, C1 = primary loading <.40, C2 = secondary loading >.30, C3 = loading difference <.20, C4 = communality <.30 | | | | | | | | | | | |  | |

| **Table D6** | |  |  |  | |  | |  |  | |  | |  | |
| --- | --- | --- | --- | --- | --- | --- | --- | --- | --- | --- | --- | --- | --- | --- |
| *Round 6 of Item Reduction: Removal of Item 18* | |  |  |  | |  | |  |  | |  | |  | |
|  |  |  |  |  | |  | |  |  | |  | |  | |
| Items | | FA 1 | FA 2 | FA 3 | h^2^ | | Decision | | | Flagging reason | | Reason to delete | |  |
| 1. I try really hard every day. | | .63 | .00 | -.08 | .39 | | Retained | | |  | |  | |  |
| 2. If I fail. I try again right away to do better. | | .80 | -.04 | -.01 | .62 | | Retained | | |  | |  | |  |
| 3. There are things I really care about. | | .35 | .39 | .04 | .37 | | Retained* | | | C1, C2, C3 | |  | |  |
| 4. I notice my thoughts and feelings. | | .58 | .22 | .04 | .49 | | Retained | | |  | |  | |  |
| 5. I give up when things are too hard. | | .37 | -.18 | .37 | .30 | | Retained* | | | C1, C2, C3 | |  | |  |
| 6. Nothing matters that much to me. | | .32 | .10 | .32 | .27 | | Retained* | | | C1, C2, C3, C4 | |  | |  |
| 7. It's OK to be scared. | | .08 | .55 | -.17 | .38 | | Retained | | |  | |  | |  |
| 8. If I think something. that doesn’t mean it’s true. | | .25 | .41 | -.06 | .31 | | Retained* | | | C3 | |  | |  |
| 11. It’s OK to feel mad. | | -.03 | .78 | .07 | .59 | | Retained | | |  | |  | |  |
| 12. If I do something bad. then I’m a bad person. | | .00 | .10 | .50 | .26 | | Retained* | | | C4 | |  | |  |
| 13. I worry a lot about stuff I did or need to do. | | .01 | -.25 | .54 | .37 | | Retained | | |  | |  | |  |
| 15. If I get angry. it means I messed up. | | -.03 | .05 | .85 | .71 | | Retained | | |  | |  | |  |
| 18. Grown-ups tell me what is important to me. | | -.25 | .12 | .23 | .09 | | Deleted | | | C1, C2, C3, C4 | | Low loading, low h^2^ | |  |
|  | *Note*. * = Flagged item, C1 = primary loading <.40, C2 = secondary loading >.30, C3 = loading difference <.20, C4 = communality <.30 | | | | | | | | | | | | | |

| **Table D7** | |  |  |  |  |  |  | |  | |  | |
| --- | --- | --- | --- | --- | --- | --- | --- | --- | --- | --- | --- | --- |
| *Round 7 of Item Reduction: Removal of Item 6* | |  |  |  |  |  |  | |  | |  | |
|  |  |  |  |  |  |  |  | |  | |  | |
| Items | | FA 1 | FA 2 | FA 3 | h^2^ | Decision | | Flagging reason | | Reason to delete | |  |
| 1. I try really hard every day. | | .67 | -.03 | -.10 | .43 | Retained | |  | |  | |  |
| 2. If I fail. I try again right away to do better. | | .78 | -.03 | .00 | .59 | Retained | |  | |  | |  |
| 3. There are things I really care about. | | .36 | .38 | .03 | .37 | Retained* | | C1, C2, C3 | |  | |  |
| 4. I notice my thoughts and feelings. | | .57 | .23 | .04 | .48 | Retained | |  | |  | |  |
| 5. I give up when things are too hard. | | .37 | -.18 | .37 | .30 | Retained* | | C1, C2, C3 | |  | |  |
| 6. Nothing matters that much to me. | | .37 | .08 | .30 | .27 | Deleted | | C1, C2, C3, C4 | | Low loading, low h^2^ | |  |
| 7. It's OK to be scared. | | .08 | .55 | -.18 | .38 | Retained | |  | |  | |  |
| 8. If I think something. that doesn’t mean it’s true. | | .22 | .44 | -.05 | .31 | Retained | |  | |  | |  |
| 11. It’s OK to feel mad. | | -.04 | .79 | .07 | .60 | Retained | |  | |  | |  |
| 12. If I do something bad. then I’m a bad person. | | .01 | .10 | .50 | .25 | Retained* | | C4 | |  | |  |
| 13. I worry a lot about stuff I did or need to do. | | -.03 | -.20 | .59 | .41 | Retained | |  | |  | |  |
| 15. If I get angry. it means I messed up. | | -.02 | .07 | .83 | .68 | Retained | |  | |  | |  |
|  | *Note*. * = Flagged item, C1 = primary loading <.40, C2 = secondary loading >.30, C3 = loading difference <.20, C4 = communality <.30 | | | | | | | | | | | |

| **Table D8** | |  |  |  |  |  | |  | |  | |  | |
| --- | --- | --- | --- | --- | --- | --- | --- | --- | --- | --- | --- | --- | --- |
| *Round 8 of Item Reduction: Removal of Item 5* | |  |  |  |  |  | |  | |  | |  | |
|  |  |  |  |  |  |  | |  | |  | |  | |
| Items | | FA 1 | FA 2 | FA 3 | h^2^ | | Decision | | Flagging reason | | Reason to delete | |  |
| 1. I try really hard every day. | | .66 | -.01 | -.09 | .43 | | Retained | |  | |  | |  |
| 2. If I fail. I try again right away to do better. | | .82 | -.03 | .02 | .66 | | Retained | |  | |  | |  |
| 3. There are things I really care about. | | .31 | .40 | .03 | .35 | | Retained* | | C2, C3 | |  | |  |
| 4. I notice my thoughts and feelings. | | .52 | .26 | .05 | .45 | | Retained | |  | |  | |  |
| 5. I give up when things are too hard. | | .36 | -.16 | .36 | .27 | | Deleted | | C1, C2, C3, C4 | | Low loading, low h^2^ | |  |
| 7. It's OK to be scared. | | .08 | .55 | -.17 | .37 | | Retained | |  | |  | |  |
| 8. If I think something. that doesn’t mean it’s true. | | .22 | .45 | -.03 | .33 | | Retained | |  | |  | |  |
| 11. It’s OK to feel mad. | | -.05 | .79 | .06 | .59 | | Retained | |  | |  | |  |
| 12. If I do something bad. then I’m a bad person. | | .01 | .10 | .49 | .25 | | Retained* | | C4 | |  | |  |
| 13. I worry a lot about stuff I did or need to do. | | -.01 | -.21 | .60 | .43 | | Retained | |  | |  | |  |
| 15. If I get angry. it means I messed up. | | -.01 | .08 | .83 | .69 | | Retained | |  | |  | |  |
|  | *Note*. * = Flagged item, C1 = primary loading <.40, C2 = secondary loading >.30, C3 = loading difference <.20, C4 = communality <.30 | | | | | | | | | | | | |

| **Table D9** | |  |  |  | |  | | |  |  | |  | |  | |
| --- | --- | --- | --- | --- | --- | --- | --- | --- | --- | --- | --- | --- | --- | --- | --- |
| *Round 9 of Item Reduction: Removal of Item 3* | |  |  |  | |  | | |  |  | |  | |  | |
|  |  |  |  |  | |  | | |  |  | |  | |  | |
| Items | | FA 1 | FA 2 | | FA 3 | | h^2^ | Decision | | | Flagging reason | | Reason to delete | |  |
| 1. I try really hard every day. | | .68 | -.08 | | -.04 | | .45 | Retained | | |  | |  | |  |
| 2. If I fail. I try again right away to do better. | | .80 | .03 | | -.04 | | .63 | Retained | | |  | |  | |  |
| 3. There are things I really care about. | | .30 | .05 | | .39 | | .34 | Deleted | | | C1, C2, C3 | | Cross-loading, poor conceptual fit | |  |
| 4. I notice my thoughts and feelings. | | .53 | .07 | | .25 | | .45 | Retained | | |  | |  | |  |
| 7. It's OK to be scared. | | .07 | -.17 | | .54 | | .37 | Retained | | |  | |  | |  |
| 8. If I think something. that doesn’t mean it’s true. | | .18 | -.04 | | .48 | | .33 | Retained | | |  | |  | |  |
| 11. It’s OK to feel mad. | | -.06 | .05 | | .78 | | .58 | Retained | | |  | |  | |  |
| 12. If I do something bad. then I’m a bad person. | | .03 | .51 | | .09 | | .27 | Retained* | | | C4 | |  | |  |
| 13. I worry a lot about stuff I did or need to do. | | .01 | .62 | | -.22 | | .45 | Retained | | |  | |  | |  |
| 15. If I get angry. it means I messed up. | | -.01 | .79 | | .07 | | .62 | Retained | | |  | |  | |  |
|  | *Note*. * = Flagged item, C1 = primary loading <.40, C2 = secondary loading >.30, C3 = loading difference <.20, C4 = communality <.30 | | | | | | | | | | | | | | |

| **Table D10** | |  |  |  |  | |  |  | |  | |  | |
| --- | --- | --- | --- | --- | --- | --- | --- | --- | --- | --- | --- | --- | --- |
| *Final 9-Item Model* | |  |  |  |  | |  |  | |  | |  | |
|  |  |  |  |  |  | |  |  | |  | |  | |
| Items | | FA 1 | FA 2 | FA 3 | h^2^ | Decisions | | | Flagging reason | | Reason to delete | |  |
| 1. I try really hard every day. | | .64 | .01 | -.08 | .42 | Retained | | |  | |  | |  |
| 2. If I fail. I try again right away to do better. | | .86 | -.03 | .03 | .72 | Retained | | |  | |  | |  |
| 4. I notice my thoughts and feelings. | | .48 | .24 | .06 | .38 | Retained | | |  | |  | |  |
| 7. It's OK to be scared. | | .05 | .61 | -.14 | .43 | Retained | | |  | |  | |  |
| 8. If I think something. that doesn’t mean it’s true. | | .16 | .52 | -.01 | .37 | Retained | | |  | |  | |  |
| 11. It’s OK to feel mad. | | -.04 | .75 | .07 | .53 | Retained | | |  | |  | |  |
| 12. If I do something bad. then I’m a bad person. | | .03 | .08 | .51 | .27 | Retained* | | | C4 | |  | |  |
| 13. I worry a lot about stuff I did or need to do. | | .02 | -.23 | .61 | .45 | Retained | | |  | |  | |  |
| 15. If I get angry. it means I messed up. | | -.01 | .07 | .81 | .65 | Retained | | |  | |  | |  |
|  | *Note*. * = Flagged item, C1 = primary loading <.40, C2 = secondary loading >.30, C3 = loading difference <.20, C4 = communality <.30 | | | | | | | | | | | | |
